# Use of thyroid hormones in hypothyroid and euthyroid patients: a 2020 THESIS questionnaire survey of members of the Czech Society of Endocrinology

**DOI:** 10.1186/s12902-022-01027-1

**Published:** 2022-05-03

**Authors:** Jan Jiskra, Jan Paleček, Roberto Attanasio, Laszlo Hegedüs, Endre V. Nagy, Enrico Papini, Petros Perros, Roberto Negro, Michal Kršek

**Affiliations:** 1 3rd Department of Medicine, 1st Faculty of Medicine, Charles University, General University Hospital, Prague, Czech Republic; 2 Scientific Committee of Associazione Medici Endocrinologi, Milan, Italy; 3 Department of Endocrinology, Odense University Hospital, Odense, Denmark; 4 Division of Endocrinology, Department of Medicine, Faculty of Medicine, University of Debrecen, Debrecen, Hungary; 5 Department of Endocrinology and Metabolism, Regina Apostolorum Hospital, Rome, Italy; 6 Department of Endocrinology, Royal Victoria Infirmary, Newcastle upon Tyne, UK; 7 Division of Endocrinology, “V. Fazzi” Hospital, Lecce, Italy

**Keywords:** Hypothyroidism, Levothyroxine, Liothyronine, Euthyroidism, Survey

## Abstract

**Background:**

Inconsistencies in the management of hypothyroidism have been reported among endocrinologists in different European countries. Aim of this study was to explore Czech endocrinologists’ use of thyroid hormones in hypothyroid and euthyroid patients.

**Methods:**

We used a web-based survey containing 32 questions regarding the use of thyroid hormones. Four-hundred thirty-two members of the Czech Society of Endocrinology received an e-mail invitation to participate in the survey.

**Results:**

We received and analysed 157 responses (112 females and 45 males) from the 432 members (36.3%). According to 99.4% of the respondents, levothyroxine (LT4) is the primary drug of choice for the treatment of hypothyroidism. Liothyronine (LT3) was used in clinical practice by 29.9% of responders. According to 90.5% of respondents, thyroid hormones may be indicated in biochemically euthyroid patients. Female physicians prescribe thyroid hormones in euthyroid infertile women with high antibody levels more frequently than male physicians (*P* = 0.003). Most Czech endocrinologists (76.4%) consider combined therapy with LT4 and LT3 in various clinical scenarios, but only 1 of 29 hypothyroid physicians (3.5%) would recommend it to their patients, and only 4 out of 128 respondents (3.1%) would consider LT3 or desiccated thyroid for themselves, if diagnosed with hypothyroidism.

**Conclusion:**

LT4 is the primary thyroid hormone used in the Czech Republic for treatment of hypothyroidism. At variance with thyroid guideline recommendations, Czech endocrinologists are quite liberal when prescribing thyroid hormones to euthyroid patients and in the use of LT4/LT3 combination treatment for hypothyroid patients with persisting symptoms.

**Supplementary Information:**

The online version contains supplementary material available at 10.1186/s12902-022-01027-1.

## Background

Hypothyroidism is the most common endocrine disease with a prevalence in the general population, when including subclinical disease, ranging from 2% in males to 8% in females [1]. Moreover, the presence of serum thyroid antibodies, marking an increased risk of development of hypothyroidism, is detected in around 11% of the general population [2]. The established treatment for hypothyroidism is levothyroxine (LT4) in tablet form [3], but recently, various LT4 formulations (soft-gel capsules or liquid solution) have become available for substitution treatment [4, 5]. Moreover, combination therapy with both LT4 and liothyronine (LT3) may be considered, though generally not recommended, in various clinical scenarios [3, 6, 7]. While the Czech Society of Endocrinology states that treatment of hypothyroidism may be managed by general practitioners [8], according to our experience, most hypothyroid patients are currently treated by endocrinologists.

The aim of this study was to explore Czech endocrinologists’ use of thyroid hormones in hypothyroid and euthyroid patients, with a special focus on the treatment of euthyroid subjects with LT4, the use of different LT4 formulations, the indications for combination therapy with LT4 and LT3, and endocrinologists’ perceptions on the cause of common symptoms among the hypothyroid patient population.

## Methods

### Survey

**Fig. 1:**
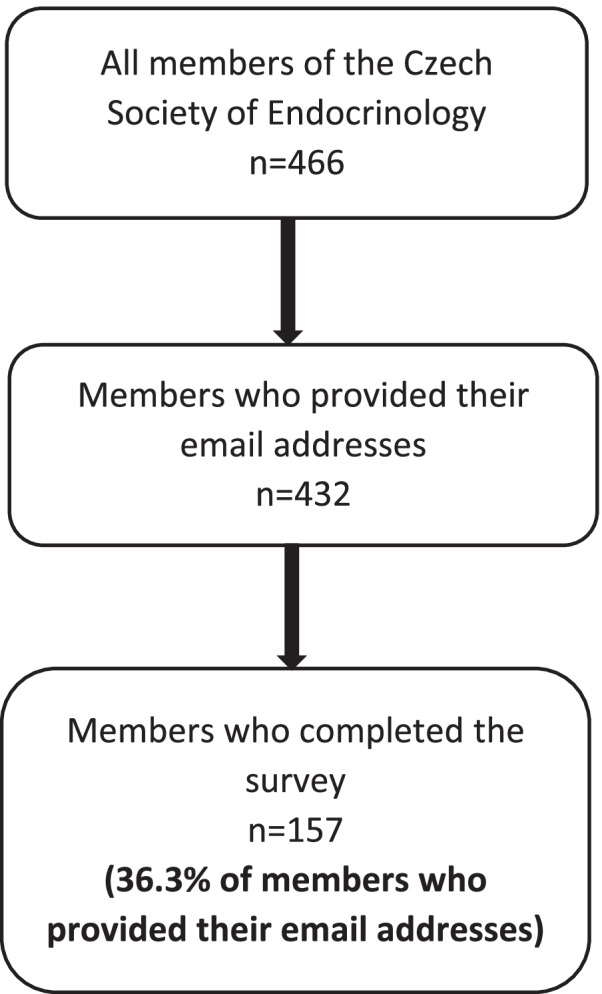
Flowchart of the Czech survey

We used a Czech language web-based survey constructed with Microsoft Forms, an open access platform that provides various question templates. The survey had 32 questions. Eight were related to the main characteristics of the respondents (age, sex, specialty, years of practice, society memberships, type of medical practice and annual number of thyroid patients seen) and 24 to the practices and preferences in the treatment of hypothyroidism. In the setting of the survey, answers to all questions were obligatory. The questionnaire was initially tested in a pilot study of Italian endocrinologists after translation into Italian, following which it underwent some revisions to its final form [9]. Some questions allowed multiple answers and some also free text. Four-hundred thirty-two members of the Czech Society of Endocrinology, who provided their e-mail addresses (Fig. 1), received an e-mail invitation on 8^th^ of August 2020 to participate in the survey. Subsequently, two reminders were sent to non-responders in September 2020 and October 2020, and the survey was closed on 7^th^ of November 2020. Survey responses were collected anonymously and stored electronically. The survey platform automatically blocked repeat submissions from the same IP address. The study is the Czech contribution to the pan-European (28 countries) survey “THESIS” (Treatment of Hypothyroidism in Europe by Specialists: An International Survey). The THESIS questionnaire was translated into Czech, and adapted from the original English version by a bilingual clinician and checked by another bilingual senior physician.

### Statistical analysis

Results are presented as absolute numbers and percentages of respondents. To evaluate the predictors of responses to questions regarding the cause of persistent symptoms in patients treated with LT4 who had already achieved normal serum TSH, multivariate regression models were constructed. The dependent variables were the responses (on a scale from 1 to 5, where 1 was strongly disagree and 5 was strongly agree) to the following question: “In most patients treated with levothyroxine who achieve normal serum TSH, persistent symptoms are due to”. The independent variables were sex, age, years in medical practice and cumulative responses to the question “Thyroid hormones may be indicated in biochemically euthyroid patients with” (sum of all “Yes” answers in each respondent). Statistical analysis was carried out using SigmaStat statistic software produced by Jandel Corporation, San Jose, California.

## Results

### Sample characteristic

Figure 1 shows a flowchart of the members of the Czech﻿ Society of Endocrinology and the respondents. Finally, we analysed 157 responses from 432 invited members of the Czech Society of Endocrinology (36.3%). The main characteristics of respondents are shown in Table 1. Most respondents were specialists in endocrinology (93.6%) and 66.9% of them had more than one medical specialty. Only 10 respondents (6.4%) had no specialty in endocrinology (6 of them were specialists in internal medicine, 3 in pediatric endocrinology and 1 in family medicine).

**Table 1:** Respondents’ characteristics

	**Number**	**Percentage of all respondents**
**Gender**		
Female	112	71.3%
Male	45	28.7%
**Age**		
20–30	0	0%
31–40	24	15.3%
41–50	73	46.5%
51–60	32	20.4%
61–70	18	11.5%
> 70	10	6.4%
**Years in clinical practice**		
0–10	13	8.3%
11–20	45	28.7%
21–30	56	35.7%
31–40	28	17.8%
> 40	15	9.5%
Specialty*		
Endocrinology	147	93.6%
Internal medicine	83	52.9%
Paediatric endocrinology	23	14.6%
Family medicine	6	3.8%
Nuclear medicine	3	1.9%
Other	18	11.5%
More than one	105	66.9%
Type of practice*		
University centre	40	25.5%
Regional hospital	33	21.0%
Private clinic	0	0%
General practice	8	5.1%
Basic researcher	2	1.3%
Specialist practice	116	73.9%
More than one	37	23.6%
**Treating thyroid patients**		
Daily	128	81.5%
Weekly	28	17.8%
Rarely	1	0.6%
**Number of treated patients**		
> 100 patients/year	126	80.2%
51–100 patients/year	24	15.3%
10–50 patients/year	7	4.5%

^*^The sum of percentages exceeds 100% because 105 respondents had more than one specialty or were employed in more than one medical unit

### Thyroid hormones available for substitution therapy

According to 156 respondents (99.4%) LT4 should be the drug of first choice for the treatment of hypothyroid patients. However, 47 respondents (29.9%) stated they also use LT3 in their clinical practice. One respondent had used desiccated thyroid in the past.

One-hundred and fifty respondents (95.5%) stated that the vast majority of their patients are dispensed the type of LT4 that they recommend.

### Absorption of various LT4 formulations, intolerance, and interfering factors

Preference of different LT4 formulations in various clinical scenarios of cases with hypothyroidism is demonstrated in Table 2. Eighty-five respondents (54.1%) stated that LT4 tablets are the least at risk of variable absorption and 57 (36.3%) expected no major change with different formulations. Ninety respondents (57.3%) would prescribe LT4 tablets in case of a diagnosis of hypothyroidism also when the patient self-reports intolerance to various foods raising the possibility of celiac disease, malabsorption, lactose intolerance, or intolerance to common excipients. Finally, 36 (22.9%) expected no major change in serum TSH levels with the use of different formulations.

**Table 2:** Preference of different LT4 formulations in various clinical scenarios of cases with hypothyroidism

	**Tablets**	**Soft-gel capsules**	**Liquid solution**	**I expect no major changes with the different formulations**
Interfering drugs may influence the stability of therapy. Which LT4 preparation is in your experience least likely to be subject to variable absorption?	85 (54.1%)	10 (6.4%)	5 (3.2%)	57 (36.3%)
Which of the following preparations of LT4 would you prescribe in case of first diagnosis of hypothyroidism when the patient self-reports intolerance to various foods raising the possibility of celiac disease, malabsorption, lactose intolerance, or intolerance to common excipients	89 (56.7%)	20 (12.7%)	12 (7.6%)	36 (22.9%)
Which of the following preparations of LT4 would you prescribe for a patient established on LT4 who has unexplained poor biochemical control of hypothyroidism?	80 (50.9%) *	18 (11.5%)	11 (7.0%)	48 (30.6%)
Which of the following preparations of LT4 would you prescribe for a patient with poor biochemical control who is unable (due to busy lifestyle) to take LT4 fasted and separate from food/drink?	76 (48.4%)	17 (10.8%)	17 (10.8%)	47 (29.9%)
Which of the following preparations of LT4 would you prescribe for a patient established on LT4 tablets who has good biochemical control of hypothyroidism but continues to have symptoms?	81 (51.6%) *	9 (5.7%)	1 (0.6%)	66 (42.0%)

^*^tablets form another manufacturer

LT4: levothyroxine

### Treatment with thyroid hormones in euthyroid patients

**Fig. 2:**
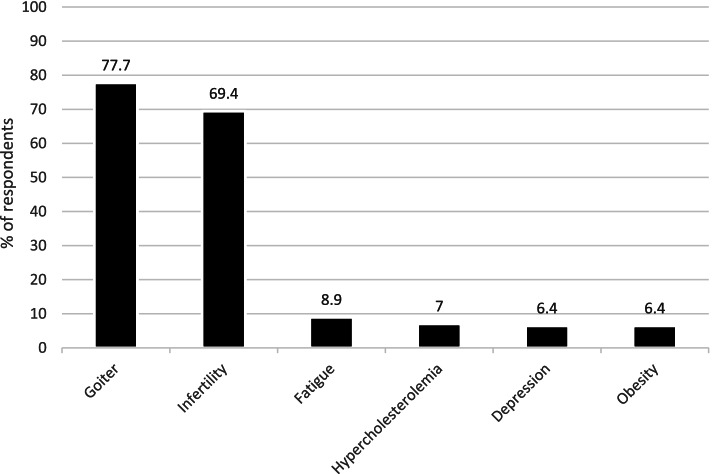
Indications of thyroid hormones in biochemically euthyroid patients considered by Czech endocrinologists

**Fig. 3:**
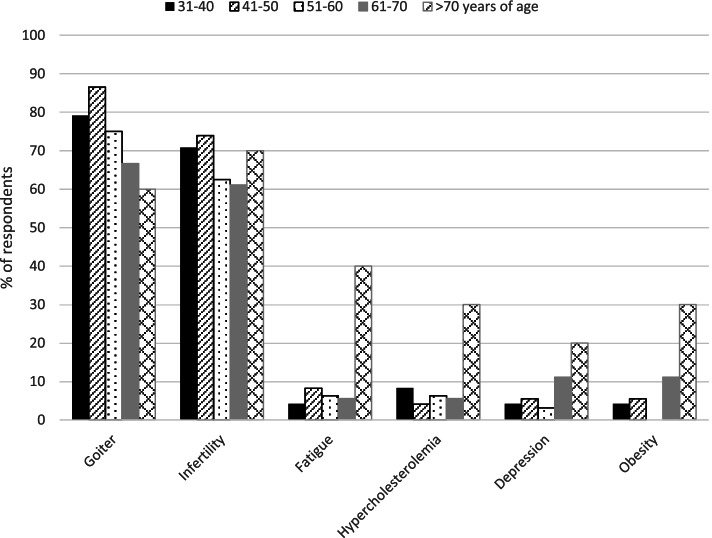
Use of thyroid hormones in euthyroid patients in different indications based on respondent’s age

One-hundred and forty-two (90.5%) respondents stated that thyroid hormones may be indicated in biochemically euthyroid patients. This varied between 6.4% in patients with obesity resistant to life-style interventions, and 77.7% in patients with a simple goiter growing over time (Fig. 2). A growing goiter and infertility in women positive for thyroid antibodies (69.4%) were the most common reasons for prescribing thyroid hormones in biochemically euthyroid patients. Less than 10% reported other indications (persistent fatigue, hypercholesterolemia, depression and/or obesity) for the prescription of thyroid hormones in biochemically euthyroid patients. Use of thyroid hormones in euthyroid patients for different indications, based on the age of the respondents, is shown in Fig. 3.

**Fig. 4:**
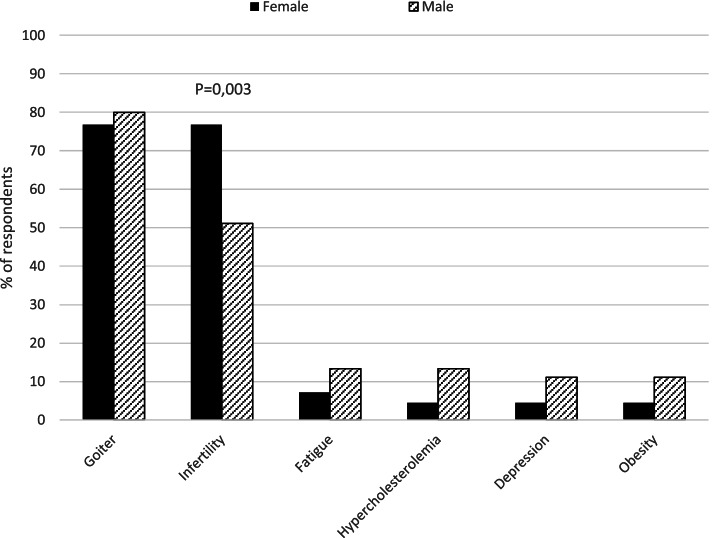
Use of thyroid hormones in euthyroid patients in different indications based on respondent’s gender

Female more frequently than male endocrinologists recommended the use of thyroid hormones in euthyroid infertile women positive for thyroid antibodies (*P* = 0.003). In contrast, male endocrinologists more often recommended the treatment in other clinical scenarios (fatigue, hypercholesterolemia, depression, and obesity). With a similar high frequency, female and male endocrinologists used thyroid hormones in euthyroid patients with a growing goiter (Fig. 4). Consistently, in a multiple logistic regression model with age, gender, years in medical practice and work environment (regional hospital, general practice, specialist practice, university centre, and basic researcher) as independent variables, female gender was associated with treatment of euthyroid infertile women with LT4 (odds ratio 2.6, 95% Confidence Interval 1.2–5.7, P = 0.02). No variable was associated with LT4 treatment in other euthyroid groups (goiter, fatigue, hypercholesterolemia, depression, and obesity).

### Treatment of patients with poor biochemical control of hypothyroidism

Only 29 (18.5%), 34 (21.7%) and 10 (6.4%) respondents would prescribe LT4 formulations alternative to tablets (liquid solution, soft-gel capsules) to patients who had unexplained poor biochemical control of hypothyroidism, were unable (due to busy lifestyle) to take LT4 fasted and separately from food/drink, or had symptoms despite good biochemical control of hypothyroidism, respectively.

### Serum TSH controls

Following start of LT4 replacement therapy, 75 respondents (47.8%) would check serum TSH after 4–6 weeks, 79 (50.3%) after 8 weeks, 1 (0.64%) after 2 weeks and 2 (1.3%) would rely only on clinical evaluation. In case of switch to a different formulation, or switch to a different LT4 tablet manufacturer, 44 respondents (28.0%) would check serum TSH after 4–6 weeks, 61 (38.9%) after 8 weeks, and 30 (19.1%) on the basis of clinical evaluation alone. Twenty-two (14.0%) stated that there is no need of TSH control after preparation change if the dosage remains the same.

### Use of dietary supplements

Ninety-nine respondents (63.1%) would recommend dietary supplements (such as selenium or iodine) in addition to thyroid hormone replacement therapy at the patient’s request or as a complementary treatment for hypothyroidism. The use of supplements was considered by 25 specialists (15.9%) in subclinical hypothyroidism and by 18 (11.5%) in presence of a coexisting autoimmune thyroiditis. A minority (15 respondents; 9.5%) stated that dietary supplements should never be used. There was no difference between the endocrinologists operating in private practice and those working in the public service about the use of dietary supplements.

### Use of combined therapy with LT4 and LT3

One-hundred and twenty respondents (76.4%) considered the use of combined therapy with LT4 and LT3 appropriate in specific clinical scenarios. Eighty-five (54.1%) would prescribe combined therapy for patients who attain normal serum TSH but still complain of symptoms suggestive of hypothyroidism. Thirty-two (20.4%) would use LT4 and LT3 therapy for a short period in patients recovering from protracted hypothyroidism, and 3 (1.9%) also in hypothyroid patients with normal serum TSH who complain of unexplained weight gain. Thirty-seven respondents (23.6%) reported that, due to the low quality of the available evidence, combined therapy should never be used.

### Reasons for persistent symptoms despite normal serum TSH in patients treated for hypothyroidism

Most respondents (113; 72.0%) reported that persistence of symptoms despite the attainment of normal serum TSH in patients treated for hypothyroidism is a rare problem concerning < 5% of patients. Only 3 (1.9%) stated that this may occur in up to 10–30% of patients. One-hundred and twenty (76.4%) believed that the frequency of these cases is decreasing or remains unchanged during the last 5 years, whereas a minority (18; 11.5%) reported seeing an increasing number of these cases.

**Fig. 5:**
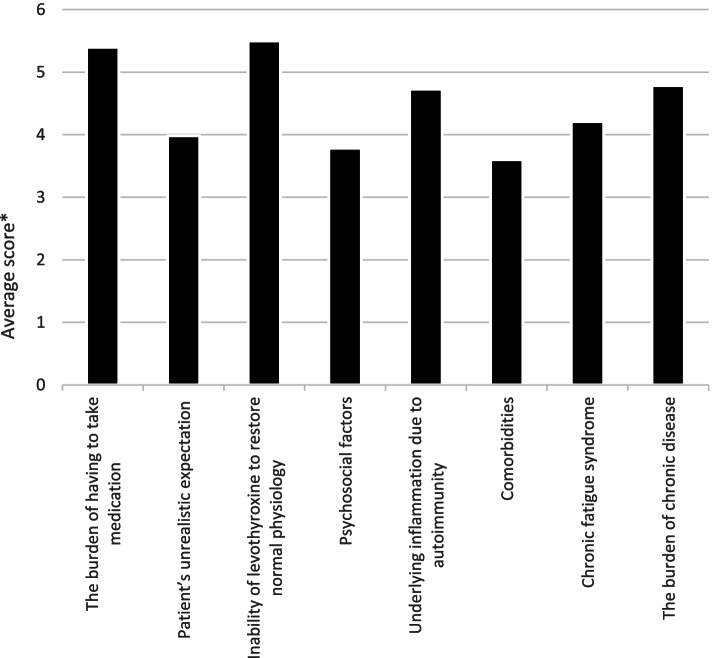
Average score of various causes of persistent symptoms in patients treated with LT4 who achieved normal serum TSH

The potential causes of persistence of symptoms in patients treated with LT4 and being biochemically euthyroid were ranked from 1 to 8, where 1 was the “least likely” and 8 was the “most likely”. The means of the answers by 157 respondents were calculated and are shown as average scores for the potential causes in Fig. 5. In a multiple regression model of the predictors of the responses to this question, young age of respondents was associated with the answers “psychosocial factors” and “patients’ unrealistic expectations”, while female sex was associated with the answers “comorbidities”, “chronic fatigue syndrome” and “patients’ unrealistic expectation”. Finally, length of medical practice was associated with the answer “patients’ unrealistic expectations” (Table 3).

**Table 3:** Multiple regression model of the predictors of reasons for persistent hypothyroid symptoms despite normal serum TSH

		**Independent variables**			
		**Sex** 1-Female 0-Male	**Age group**	**Years in medical practice**	Thyroid hormones may be indicated in biochemically euthyroid patients with…*
**Dependent variables**	**Inability of levothyroxine to restore normal physiology**	NS	NS	NS	NS
	**Psychosocial factors**	NS	β-coeff. = -0.278 Std. Error = 0.128 *P* = 0.031	NS	NS
	**Comorbidities**	β-coeff. = 0.312 Std. Error = 0.129 *P* = 0.017	NS	NS	NS
	**Chronic fatigue syndrome**	β-coeff. = 0.353 Std. Error = 0.165 *P* = 0.033	NS	NS	NS
	**Patient unrealistic expectation**	β-coeff. = 0.319 Std. Error = 0.160 *P* = 0.048	β-coeff. = -0.355 Std. Error = 0.138 *P* = 0.011	β-coeff. = 0.383 Std. Error = 0.138 *P* = 0.006	NS
	**Presence of underlying inflammation due to autoimmunity**	NS	NS	NS	NS
	**The burden of chronic disease**	NS	NS	NS	NS

^*^ …depression resistant to anti-depressant medications; female infertility with high level of thyroid antibodies; simple goitre growing over time; unexplained fatigue; obesity resistant to life-style interventions; severe hypercholesterolemia, as a complementary treatment (sum of all “Yes” answers in each respondent)

*TSH* Thyroid stimulating hormone, *NS* Not significant, *P* Level of significance

### Respondents with diagnosis of hypothyroidism

Twenty-nine respondents (18.5%) had been diagnosed with hypothyroidism and treated with thyroid hormones, and 3 of them (10.3%) stated that they experienced excessive fatigue. Of these 29, none had tried preparation of desiccated thyroid and 4 (13.8%) had tried combined therapy with LT4 and LT3. Among the latter 4 respondents, 2 preferred LT4 and one reported no difference between LT4 and combined LT4-LT3 therapy. Only one out of 29 hypothyroid physicians (3.5%) would recommend LT3 to their patients. Similarly, only 4 out of 128 respondents (3.1%) would consider LT3 or desiccated thyroid for themselves, if diagnosed with hypothyroidism.

## Discussion

Hypothyroidism is a common condition and thyroid hormones are among the most frequently prescribed drugs [10]. For these reasons the appropriate use of replacement therapy is of great importance in terms of both clinical efficacy and financial costs. The Czech Society of Endocrinology recommends following the guidelines for management of hypothyroidism published by the American Thyroid Association [3], the European Thyroid Association [11], and the national guidelines for management of hypothyroidism [8]. Although LT4 is recommended as the primary drug of choice, well-conducted prospective randomized trials comparing efficacy of various LT4 formulations (tablets, desiccated thyroid, soft-gel capsules, liquid solution, etc.), and combined therapy with LT4 and LT3 are limited [6, 12]. Furthermore, there is variable availability of various LT4 formulations and LT3 across the European countries. This leads to regional variations in the treatment of hypothyroidism among European countries, but also among physicians in the same country [13]. Questionnaire surveys can explore differences in clinical practice and compare the real-world practice to the recommendations provided by guidelines. Interestingly, we were able to compare the responses from Czech endocrinologist with the results of the published THESIS surveys carried out in Denmark [14], Italy [9], Romania [15], Bulgaria [16], and Poland [17].

### Surveyed physicians

One-hundred and fifty-seven (112 females and 45 males) of the 432 members of Czech Endocrine Society who provided their e-mail addresses (36.3%) filled in the questionnaire (Fig. 1). Seventy-three percent of the respondents were female endocrinologists which approximately reflects the proportion of female members of the Czech Society of Endocrinology (67% females vs. 33% males). It is in accordance with Bulgarian [16], Romanian [15], Polish [17] and Italian [9] surveys. On the contrary, only 44% of the respondents were females in Danish survey [14].

As 93.6% of respondents were specialists in endocrinology in our survey, the answers to all questions reflect predominantly the view of endocrinologists.

The response rate (36.3%) is similar to that of previously published THESIS surveys from Denmark (31.2%) [14] and Italy (39.3%) [9], but slightly lower than those from Romania (42.2%) [15] and Poland (54.6%) [17]. The sample may be considered as representative because most respondents (99.4%) stated that they managed hypothyroid patients on a regular basis (daily or weekly). It has been suggested that the relationship between the survey response rate and non-response bias is small [18]. Despite this, we have tried to assess non-response bias using two different approaches [19, 20]. One approach was to assess the influence of measured variables on response rate, and another was to compare with the population. Neither of these approaches showed systematic bias, so it seems that this is a representative sample.

### Thyroid hormones available for substitution therapy

At the time of the survey, two LT4 tablet brands, none LT4/LT3 combined tablet brand and one LT3 tablet brand were licensed in Czech Republic. No formulation of soft-gel capsules, LT4 liquid solution, or preparations of desiccated thyroid were available in Czech Republic at the time of the survey.

In accordance with current guidelines [3], almost all respondents considered LT4 the drug of choice for treatment of hypothyroidism. Despite lack of evidence [6], one third of respondents stated that they had also used LT3 in combination with LT4 in their clinical practice. Only one respondent had experience with desiccated thyroid, and none had ever used soft-gel capsules or liquid solution with LT4. This information is in line with that obtained from Italian [9], Romanian [15], Bulgarian [16], and Polish [17] endocrinologists. In contrast, Danish physicians tended to prescribe more frequently LT4 + LT3 combination therapy (58.6%) [14] as compared to the Czech physicians.

Most Czech respondents stated that tablets are the least likely to be subject to variable absorption and intolerance/interference and that they expected no major changes with different LT4 formulations (90.4% and 82.2%, respectively). Consistently, less than one quarter of respondents would prescribe LT4 formulations different from tablets in cases with unexplained poor control of hypothyroidism or in patients who are unable to take LT4 fasted and separately from food/drink or have persistent symptoms despite good biochemical control of hypothyroidism. These answers are in accordance with those obtained from most other European surveys but are quite dissimilar to those provided by the Italian endocrinologists who are probably more familiar with the various LT4 formulations because most of the clinical studies on liquid and soft gel capsules have been performed in Italy [9]. The answers from Czech physicians referring to the different LT4 formulations may be biased by the fact that neither soft-gel capsules, nor LT4 liquid solution, or preparations of desiccated thyroid were available in the Czech Republic at the time of the survey.

### Treatment with thyroid hormones in euthyroid patients

According to current evidence, depression, obesity, unexplained fatigue, and hypercholesterolemia are not regarded as indications for thyroid hormone treatment in euthyroid subjects [21–23]. Interestingly, most Czech endocrinologists still consider the use of thyroid hormones for patients with euthyroid goiter. This is not in agreement with the evidence demonstrating that most patients with goiter do not benefit from LT4 suppressive therapy [24] and with the recommendations of current guidelines [25]. Although treatment with LT4 in doses leading to suppression of TSH could modestly reduce goiter size [24, 26] and the risk of malignancy in a thyroid nodule increases with serum TSH concentrations even within the normal range [27], the modest and inconstant favourable effects on thyroid volume disappear after treatment withdrawal [24]. In addition, the potentially adverse cardiovascular [28] and bone effects [29], as well as the possible influence on psychiatric morbidity [30], probably outweigh the benefits and is associated with excess mortality [31]. These considerations require action in order to improve adherence to the available scientific evidence.

Positive thyroid antibodies in euthyroid women have been associated with infertility and risk of abortions and preterm deliveries [32]. However, at variance with earlier studies [33], the recent large studies found that LT4 intervention had no impact on the rate of miscarriage, preterm delivery, and live births in euthyroid thyroid antibody positive women [34, 35].

Despite lack of evidence, most Czech endocrinologists (90.5%) believe that thyroid hormones may be indicated in biochemically euthyroid patients and would use these in different clinical situations. Interestingly, this is a much higher rate compared to Danish (48.7%) [14], Italian (46.6%) [9], Romanian (52%) [15], Polish (74.2%) [17], and Bulgarian (66.7%) [16] endocrinologists. According to the Czech endocrinologists, a growing goiter (77.7%) and positive thyroid antibodies in female infertility (69.4%) are the most frequent reasons for treatment. Notably, older endocrinologists (> 70 years) would recommend thyroid hormones more often than the younger endocrinologists also in cases different from goitre and infertility. The fact that female more often than male endocrinologists recommend thyroid hormones in infertile women with positive thyroid antibodies (Fig. 4) remains unexplained, as does the fact that, overall, the treatment of euthyroid patients with LT4 is so widely considered among Czech endocrinologists.

### Serum TSH controls, use of dietary supplements

In case of serum TSH monitoring after the start of LT4 replacement therapy the vast majority (98.1%) of respondents would follow current guidelines [3, 8, 11] and re-check TSH after 4–8 weeks.

In total 90.5% of Czech endocrinologists would prescribe dietary supplements (such as selenium or iodine) at the patient’s request or as a complementary treatment of subclinical hypothyroidism and/or autoimmune thyroiditis. This management approach appears to be a widespread practice even though the iodine status in the Czech Republic is sufficient in view of obligatory universal salt iodization. In addition, the evidence of clinical efficacy of selenium in improving the clinical course of chronic thyroiditis is poor [36], even if selenium in a few studies was reported to decrease thyroid autoantibody levels [37].

### Use of combined therapy with LT4 and LT3

The European Thyroid Association (ETA) suggests that LT4 + LT3 combination therapy might be considered as a short-term therapeutic approach in those LT4-treated and proven compliant hypothyroid patients who have persistent hypothyroid-like symptoms despite serum TSH values within the reference range. These patients should preliminarily have given support to deal with the chronic nature of their disease and coexistent autoimmune diseases should have been ruled out. ETA also suggests an LT4/LT3 dose ratio between 13:1 and 20:1 by weight and recommends the daily LT3 dose should be divided in two doses [38]. However, evidence supporting this practice remains limited [6]. The main disadvantage of LT4 + LT3 combined therapy used in conventional tablet preparations is undesirable fluctuation of LT3 serum concentration which could be associated with adverse cardiovascular and bone effects [28, 29]. The use of slow release LT3 preparations is desirable in future trials to achieve physiological LT3 levels [6]. Moreover, LT4 + LT3 combination therapy is not recommended in pregnant women because of a risk of foetal hypothyroxinemia [39]. Despite the low-quality evidence, most Czech endocrinologists (76.4%) support that combined therapy with LT4 and LT3 may be considered in specific clinical scenarios. About one-fourth of Czech endocrinologists (23.6%) never use combination therapy, which is similar to Romanian (22.4%) [15], and Polish (28.4%) [17], but lower than Danish (71.1%) [14], Italian (41.4%) [9], and Bulgarian (40.0%) [16] endocrinologists. Consistent with other European surveys, the most common indication for combined LT4 + LT3 therapy was presence of symptoms suggestive of hypothyroidism in patients who attain normal serum TSH.

### Reasons for persistent symptoms despite normal serum TSH in patients treated for hypothyroidism

Mitchel et al. recently published that more than three quarters patients with treated hypothyroidism reported dissatisfaction with treatment [40]. In our study, more than 2/3 of respondents assumed that persistent symptoms despite normal serum TSH in patients treated for hypothyroidism is a rather rare problem, which is consistent with Polish [17], Romanian [15], and Bulgarian [16] surveys. Also, most Czech endocrinologists observed fewer such cases, or no change over the past 5 years. In contrast, most Danish respondents (58.6%) stated that the prevalence of such cases is increasing [14].

Four principal hypotheses may explain the enigma of persistent symptoms in hypothyroid patients treated with LT4. The “low tissue liothyronine hypothesis”, the “somatic symptom and related disorders hypothesis”, the “autoimmune neuroinflammation hypothesis” and the “comorbidities and psychosocial hypothesis” [41]. In our study, “inability of levothyroxine to restore normal physiology” and “burden of having to take medication” were the most likely causes for persistent symptoms considered by Czech endocrinologists, whereas “comorbidities”, “psychosocial factors”, and patients’ unrealistic expectations were the least likely. This is in contrast to the finding in the Danish survey [14], where only a minority stated that symptom persistence might be due to LT4’s inability to restore normal physiology, indicating that Danish endocrinologist adhere to the available scientific evidence more consistently than the Czech.

### Respondents with diagnosis of hypothyroidism

Interestingly, although most (76.4%) Czech endocrinologist stated that combined therapy with LT4 and LT3 may be considered in various clinical scenarios, only 3.5% of respondents, who themselves have a diagnosis of hypothyroidism, would recommend LT3. Similarly, only 3.1% of respondents would consider LT3 for themselves if diagnosed with hypothyroidism. This trend was also evident in other national THESIS surveys [9, 14–17]. A reason for this inconsistency could be an effort of the physicians to give in to the demands of their patients but having some concern about LT3 side effects and doubts about efficacy. The aggregate data from the 28 national investigations will offer power to analyse this in greater detail.

### Strengths and limitations

As strengths of our study, the vast majority (93.6%) of respondents were specialists in endocrinology and included the key opinion leaders. Moreover, the questionnaire was translated into Czech, which is expected to have limited bias in favour of English-speaking responders and to have increased response rate.

As limitations, we did not exclude during the invitation process members who were not eligible for the survey, e.g., endocrinologists who did not routinely manage hypothyroid patients. The relatively low response rate (36.3%) was similar to that attained in surveys in Denmark (31.2%) [14], and Italy (39.3%) [9], and questions generalizability of our results to the entire population of treating physicians. However, the fact that we did not exclude members who were not eligible for the survey suggests that our response rate is de facto underestimated. Although a care was taken to block duplicate responses from the same IP address, the possibility of duplicate responses submitted from different IP addresses cannot be excluded, however we consider that to be highly unlikely, and we deem this to be a negligible bias in this kind of work.

## Conclusions

In the Czech Republic, LT4 is the primary thyroid hormone used for substitution therapy in hypothyroidism. The prevalence of persistent symptoms in biochemically euthyroid LT4 treated patients was reported to be rather low and its prevalence appears to be declining. At variance with recommendations in major guidelines, Czech endocrinologists frequently prescribed thyroid hormones to euthyroid patients. Though robust evidence is lacking, most of the specialists also considered the use of combined LT4 + LT3 therapy. These data demonstrate an incomplete adherence of Czech specialists to evidence-based recommendations and suggest the need for action to modify practice attitudes toward the treatment of hypothyroidism. A significant discrepancy between hypothyroid endocrinologists’ practice in the use of combination treatment for their patients or themselves merits further study but could be due to pressure by dissatisfied patients.

## Supplementary Information

**Additional file 1.** 

## Data Availability

All data generated or analysed during this study are included in this published article and its supplementary information files.

## Abbreviations

ATA: American Thyroid Association
ETA: European Thyroid Association
LT3: Liothyronine
LT4: Levothyroxine
THESIS: Treatment of Hypothyroidism in Europe by Specialists: An International Survey
TSH: Thyroid stimulating hormone
